# Modulation of Motor Cortex Excitability by Physical Similarity with an Observed Hand Action

**DOI:** 10.1371/journal.pone.0000971

**Published:** 2007-10-03

**Authors:** Marie-Christine Désy, Hugo Théoret

**Affiliations:** Département de psychologie and Hôpital Sainte-Justine, Université de Montréal, Montréal, Canada; Harvard Medical School, United States of America

## Abstract

The passive observation of hand actions is associated with increased motor cortex excitability, presumably reflecting activity within the human mirror neuron system (MNS). Recent data show that in-group ethnic membership increases motor cortex excitability during observation of culturally relevant hand gestures, suggesting that physical similarity with an observed body part may modulate MNS responses. Here, we ask whether the MNS is preferentially activated by passive observation of hand actions that are similar or dissimilar to self in terms of sex and skin color. Transcranial magnetic stimulation-induced motor evoked potentials were recorded from the first dorsal interosseus muscle while participants viewed videos depicting index finger movements made by female or male participants with black or white skin color. Forty-eight participants equally distributed in terms of sex and skin color participated in the study. Results show an interaction between self-attributes and physical attributes of the observed hand in the right motor cortex of female participants, where corticospinal excitability is increased during observation of hand actions in a different skin color than that of the observer. Our data show that specific physical properties of an observed action modulate motor cortex excitability and we hypothesize that in-group/out-group membership and self-related processes underlie these effects.

## Introduction

The contribution of the frontoparietal mirror neuron system (MNS) to action understanding has been well documented in the human brain (see [1]). The inferior parietal lobule (IPL) and the inferior frontal gyrus (IFG) appear to be the main components of a system matching the observation and execution of actions. At the motor cortex level, passive observation of hand actions increases M1 excitability, presumably reflecting mirror neuron activity originating from premotor areas [2]. Using transcranial magnetic stimulation (TMS), Fadiga and collaborators [3] have shown that observation of hand grasping movements significantly increases the amplitude of TMS-induced motor evoked potentials (MEPs). Corticospinal facilitation has also been found to be specific to the muscles involved in the observed action [3]–[5] and to follow the temporal structure of the observed movement [6]. Furthermore, the pattern of increased MEP amplitudes varies with laterality of the observed body part [7], observer posture [8], as well as orientation of the observed hand [5]. Importantly, paired-pulse TMS evidence suggests cortical involvement in the modulation of M1 excitability during action observation [4].

Despite rapidly accumulating data detailing MNS properties in the healthy human brain, little is known about how physical similarity with an observed body part modulates M1 activity during action observation. Molnar-Szakacs and collaborators [9] have recently shown increased motor cortex responses during observation of culturally-relevant hand gestures performed by in-group members. Specifically, it was found that M1 excitability was greater when participants observed gestures performed by an actor of the same ethnic background compared to an out-group member, in line with imaging data showing that same-race face processing is associated with increased activity in the fusiform gyrus [10] and amygdala [11]. This is supported at the behavioral level by the fact that stronger social projection attitudes usually occur towards in-group members whereby the expectation of similarity between self and others is increased [12].

Following on that, it may be hypothesized that in addition to in-group/out-group membership, the relationship between motor cortex excitability and physical similarity with an observed action is related to self-other representation. Indeed, the notion of physical similarity inevitably refers to one's own body, such that specific self-referential neural mechanisms may modulate MNS responses to observed actions. Neuroimaging data suggest that the network of cortical areas subserving self-face recognition overlaps with the frontoparietal MNS system. For example, activity in the right IPL and IFG increases when participants are presented with morphed faces containing increasing levels of ‘self’ [13] and transient disruption of the right IPL with TMS significantly impairs performance on a self-other discrimination task [14]. At the motor cortex level, Keenan and collaborators [15] have shown that observation of faces contaning elements of one's own face is associated with increased motor cortex excitability compared to faces containing elements of a familiar individual's face. A subsequent study extended these data by showing increases in motor cortex excitability during observation of self-faces in the absence of conscious awareness [16]. As it relates to hand actions, however, few studies have addressed the issue of self/other representation and the MNS at the motor cortex level. Patuzzo and collaborators [17] found no evidence for differential modulation of corticospinal excitability during observation of self- and non-self finger movement. In another study [18], corticospinal excitability was assessed with TMS during the ‘rubber-hand illusion’, which can result in illusory ownership of an external body part [19]. Corticospinal excitability was significantly *increased* when subjects attributed an observed action to another person whereas attribution of the same action to self suppressed excitability [18]. This led Schutz-Bosbach and collaborators [18] to formulate the ‘social differnentiation’ hypothesis, suggesting a specific MNS involvement in *differentiating* self from other rather than *equating* them.

In the present study, we addressed the question of physical similarity with an observed body part and its effect on motor cortex excitability by having healthy participants passively observe finger movements that were either similar or dissimilar to self in terms of sex and skin color. According to the in-group/out-group hypothesis, observation of hand actions performed by similar others should lead to increased motor cortex activity during observation of hand actions performed by similar others. Conversely, the social differentiation hypothesis would predict increased motor cortex excitability during observation of dissimilar others if self-other representation is involved in similarity judgements. Furthermore, since it has been suggested that empathy differences may explain the differential pattern of MNS responses observed in men and women in some studies [20], [21], an empathy questionnaire was administered to all participants.

## Methods

### Subjects

Forty-eight healthy right-handed volunteers (12 white females, 12 black females, 12 white males and 12 black males) aged 18 to 35 years old participated in the study. All participants gave written informed consent and the study was approved by the local institutional review board of Université de Montréal.

### Stimuli

**Figure 1:**
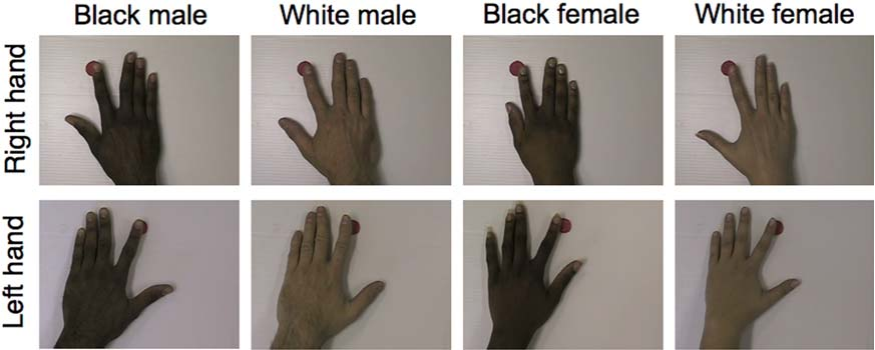
Participants observed index finger movements performed by a hand varying on color (black, white) and gender (male, female). The index finger moved toward the red dot at a pace of 1 Hz.

TMS-induced MEPs from the first dorsal interosseus (FDI) muscle were recorded while participants passively viewed 4-second movie clips on a 17” high-resolution computer screen set at eye level at a distance of one meter. Participants observed an index finger moving towards a red dot at a pace of 1 Hz from the back of an outstretched hand facing away from the observer [7]. Four different hands were randomly presented to each participants *i*) white female; *ii*) black female; *iii*) white male; *iv*) black male (Figure 1). To account for lateralization effects [7], participants only observed actions performed by the hand contralateral to the stimulated hemisphere. Each movie clip was presented 16 times and a single TMS pulse was delivered during each clip presentation between 2.5 and 3.5 seconds after the start of the movie. Eight TMS pulses were delivered at the beginning and end of the experiment to establish a baseline level. Following the TMS experiment, a questionnaire assessing empathy was administered to all participants. The Empathy Quotient (EQ; [22]) is a short, self-administered questionnaire comprising 40 items tapping empathy and 20 filler items.

### Transcranial magnetic stimulation

TMS was delivered over the left (24 participants) or right (24 participants) hemisphere with a commercially available 80-mm figure-of-eight coil and a Magpro ×100 magnetic stimulator (Medtronic, Minneapolis, USA). The current waveform was biphasic and the orientation of the stimulation coil was 45° from the midline with the handle pointing backwards. All stimulation was performed at an intensity adjusted to evoke MEPs of approximately 1mV peak-to-peak amplitude at the optimal scalp site for induction of MEPs in the FDI. The electromyographic signal was recorded using a PowerLab 4/30 system (ADInstruments, Colorado Springs, USA), filtered with a band pass of 20–1000 Hz and digitized at a sampling rate of 4 KHz. Data were stored on a computer for off-line analysis. MEPs were recorded and analyzed using Scope software (ADInstruments, Colorado Springs, USA).

### Data analysis

Peak-to-peak amplitudes of the collected MEPs were measured and averaged for baseline and each of the four experimental conditions. Percent change from baseline was calculated for each condition to account for the large interindividual variability in MEP size [23]. A 2×2×2×2×2 repeated measures ANOVA was performed with *Hemisphere, Sex* and *Skin color* as between subjects factors and *Color of the hand* (hand color) and *Sex of the hand* (hand sex) as within-subjects factors.

## Results

**Figure 2:**
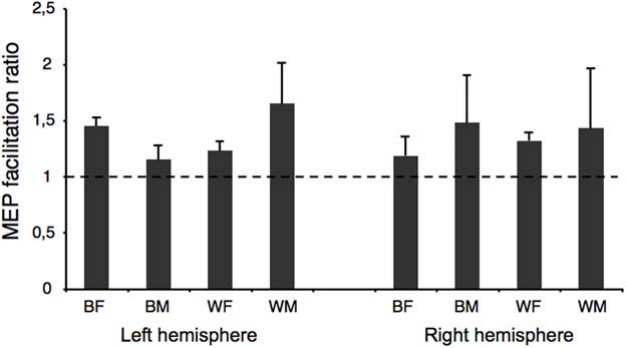
Facilitation ratios of all action conditions compared to baseline (mean and standard error). In all groups, passive observation of finger movements increases motor cortex excitability compared to baseline. BF: black female; BM: black male; WF: white female; WM: white male.

**Figure 3:**
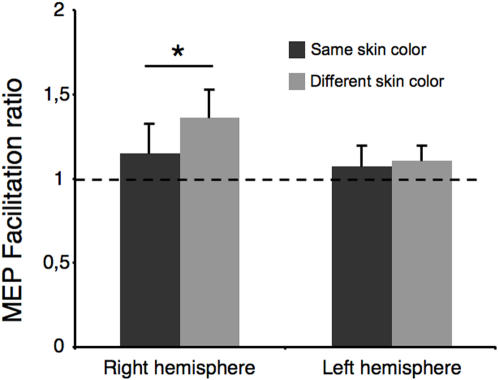
Facilitation ratios for the *skin color* comparison in female participants. In the right hemisphere, observing hand movements performed by a hand of a different color from that of the participant produces more motor cortex facilitation than observation of a hand of similar color.

Preliminary analysis of the raw data revealed no significant difference between groups in the level of corticospinal excitability during observation of finger movement (Figure 2). A repeated measures 5-way ANOVA revealed no significant main effect of *Sex, Skin color* or *Hemisphere*. However, there was a significant interaction between all 5 factors (*F* = 6.50, *P* = 0.015). For the right hemisphere, there was no significant main effect, but the interaction between *Sex, Skin color* and *Handcolor* was significant (*F* = 6.40, *P* = 0.019). In women, there was a significant interaction between *Skin color* and *Hand color* (*F* = 14.07, *P* = 0.004), which was explained by the fact that in both white and black female participants, observation of a finger movement executed by a hand in a different skin color produced greater corticospinal faciliation than observation of a hand in a similar color (Figure 3). No significant effect was found for male participants in the right hemisphere. In the left hemisphere, there were no main effects or interactions with *Handcolor* or *Handsex*.

**Figure 4:**
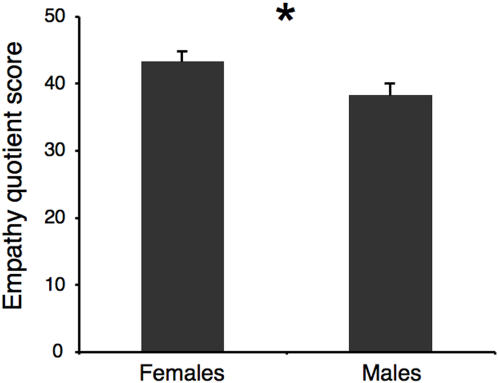
Mean empathy quotient score for male and female participants. *: p<0.05.

Scores on the EQ scale were submitted to ANOVA with *Sex* and *Skin Color* as factors. A significant effect of *Sex* was found (Figure 4; *F* = 4.27, *P* = 0.045), with women scoring higher than men. To determine the relationship between sex differences in empathy and motor cortex responses to hand actions, correlational analyses were performed. There was no correlation between mean MEP size (averaged for all conditions) and scores on the EQ for both men (*r* = 0.03, *P* = 0.905) and women (*r* = 0.18, *P* = 0.401). Correlational analysis was also performed between scores on the EQ and MEP size when participants observed hand actions executed by a hand of a different color. Again, there was no significant correlation for both men (*r* = −0.24, *P* = 0.910) and women (*r* = 0.21, *P* = 0.333).

## Discussion

The present study set out to explore the relationship between the MNS for action and physical properties of an observed body part. Consistent with previous findings (see [2]), passive observation of hand actions facilitated corticospinal excitability across all conditions. This facilitation was greater in female participants when the model hand was of a different skin color than that of the observer and the effect was restricted to the right hemisphere. Although women scored significantly higher than men on an empathy scale, the absence of correlation with corticospinal excitability does not support the hypothesis that MNS sex differences are related to empathy.

In-group/out-group perception may account for the pattern of motor cortex responses observed in the present study. A strong bias favouring members of one's in-group has consistently been reported [24] whereby similarity beetwen self and other is presumed to be increased within the in-group [12]. At the brain level, numerous studies have reported patterns of race-related amygdala activations reflecting in-group/out-group membership (see [25] for review). Hart and collaborators [26] reported prolonged amygdala habituation rates during the observation of faces belonging to the racial out-group compared to the in-group. It was later reported in white participants that black faces were associated with greater amygdala activation than white faces when the target stimuli were prevented from entering conscious awareness [11]. When target faces were consciously perceived, the race effect occurred in prefrontal and cingulate cortex, suggesting inhibition and control of negative racial attitudes [11], [27]. Based on findings of increased motor cortex excitability during observation of culture-specific gestures performed by ethnic in-group members, Molnar-Szakacs et al. [9] have suggested that the MNS is “differentially sensitive to in-group versus out-group members”. Our data are in general agreement with this statement but differ on one important aspect: whereas in-group membership was associated with increased motor cortex excitability in the Molnar-Szakacs study, we found increased motor cortex activations during observation of actions performed by a hand of a *different* color than that of the observer.

Increased excitability during observation of movements performed by racial out-group members may seem counterintuitive. Indeed, familiarity with an observed action has been shown to increase activity in MNS areas. For example, when experts in a specific dance style observe their own dance style performed by others, there is greater activity within the classical MNS areas than during observation of an unpracticed dance [28], even if the dance has only recently been learned [29]. Furthermore, observation of actions performed by conspecifics results in greater MNS activation than actions performed by non-conspecifics [30], again suggesting that familiarity and the existence of a common motor repertoire increases motor resonance. As such, the current findings indicate that in-group/out-group membership may not entirely explain the pattern of responses found in primary motor cortex in response to hand actions presenting varying levels of physical similarity with the observer. Perception of similarity with a physical characteristic present in another individual inevitably involves self/other representation. To achieve a correct determination of in-group/out-group membership during action observation, one must necessarily compare self and other at a physical level. Furthermore, there is considerable evidence suggesting preferential involvement of the right hemisphere in the self/other concept (see [31]–[32]). Behavioral, imaging and lesion data point to a network of right hemisphere areas subserving self-recognition that include frontal and parietal cortex [32], both important components of the MNS.

Interestingly, it has recently been suggested that mirror matching may underlie ‘social differentiation’, such that the motor system comprises distinct representations of self and other [18]. In this view, mirror mechanisms at the motor cortex level are not agent-neutral but rather depend on who is performing the action. In the ‘rubber hand illusion’, synchronous stroking of a rubber hand and a participant's own unseen hand causes the fake hand to be perceived as part of one's own body [19]. Using this manipulation of body ownership, Schütz-Bosbach et al. [18] have shown that motor cortex facilitation induced by action observation is greater when the action is attributed to *another* person. Motor cortex excitability increases associated with non-self stimuli have also been reported during the reading of self descriptive personality-trait words [33]. In a group of healthy participants, it was found that reading words rated as ‘never’ self characteristic showed greater facilitation than words rated as ‘always’ characteristic [33]. Along the same lines, imagination of body movement performed by another agent produces greater corticospinal facilitation than imagination of one's own body movement [34]. Also in agreement with the current data, Cheng and collaborators [20] have reported stronger motor cortex activation in female participants during observation of hand actions performed by a male actor, suggesting an “opposite-gender response, that is, female participants responded stronger to displayed male hands”. The present data are in general agreement with the ‘differentiation’ hypothesis since corticospinal facilitation resulting from the observation of index-finger movement is greater when the model hand is of a different skin color than that of the observer. This suggests that observation of an action that is peformed by an agent that is clearly different from the observer preferentially activates the motor cortex node of the MNS although it is still significantly activated by observation of a similar other. Following this reasoning, the lack of differential modulation induced by observation of male and female hands could be explained by the fact that skin color is a much more salient visual feature than gender when looking at hand actions. Indeed, as seen in Figure 1, *gender* of the observed hand is somewhat ambiguous compared to *color* of the observed hand, which is unmistakable.

It must be mentioned that the association between physical similarity with an observed hand and self/other representation remains speculative. Contrary to the differentiation hypothesis, corticospinal excitability increases when people are presented with faces containing elements of their own face [15] and imaging studies have reported increased activity in frontoparietal areas during self recognition [13]. It would be necessary to directly compare the effects of observing one's own hand to that of a similar/dissimilar individual to determine the contribution of self-related mechanisms to the motor cortex excitability effects reported here. It must be pointed out, however, that Patuzzo et al. [17] found no differences in corticospinal excitability during observation of self and non-self finger movement. The same study reported that many participants misattributed ownership of the observed hand, suggesting that contrary to faces, it is much harder to distinguish one's own hand from that of a stranger. A clear distinction between self and other, such as skin color, may be required for the motor cortex to respond differentially to stimuli varying in similarity with one's own body. Further studies are needed to directly compare the effects of similarity and self/other perception on motor cortex excitability using both hand and face stimuli.

One may wonder why the observed effect was limited to female participants. There is no evidence for gender differences in racial attitudes [35] and imaging studies of race perception have not reported a gender effect in activation patterns [25]. Gender differences in MNS activity have been reported in two recent studies. Cheng et al. [20] first showed that mu rhythm suppression occuring in sensorimotor cortex during action observation was significantly greater in women. Subsequently, the same group reported stronger modulation of spinal excitability in response to the observation of moving feet in female participants [21]. The authors interpreted results from both studies as indicative of empathy differences at the gender level. This hypothesis is based on the well-known sex differences in empathy [36] and the suggested role of mirror-matching mechanisms in the cascade of neural events leading to empathy [37]. We specifically addressed the issue of female superiority in empathy and MNS activity by having all participants complete a self-report empathy questionnaire. In accordance with previous findings, female participants scored significantly higher than men on the EQ [22]. However, there was no significant correlation between EQ score and motor cortex excitability in men or women, suggesting that empathy levels do not significantly contribute to the modulatory effect of action observation on corticospinal excitability. It is difficult at this point to explain why gender effects appear in some studies of MNS function and not in others. Indeed, most investigations of the MNS have failed to report sex differences related to action observation [1] and a previous TMS study using similar stimuli as those used in the present study found no effect of sex on corticospinal excitability [7]. Discrepancies between studies as they relate to sex differences may be explained by baseline effects. In the Cheng et al. [20] study, a moving dot was used as the control condition. As the authors themselves point out, it has been shown that viewing a moving dot can modulate motor cortex activity [38], raising the possibility that it is the control stimulus that is treated differently by men and women.

It remains that the gender effect found here is puzzling. One may speculate that known gender differences in self-concept [39] are somehow related to the differential pattern of motor cortex responses found in the present study. Specifically, it may be argued that the gender specificity of particular MNS-related phenomena may partly be explained by the presence of different ‘self-construals’ in men and women [39]. Self-construal is defined as an “individual's sense of self in relation to others” [40]. An *independent* self-construal is associated with a reduced influence of others on the self (others are excluded from the self) whereas the *interdependent* self-construal is associated with the assimilation and inclusion of others to the self [41]. Importantly, it has been shown that non-conscious mimicry, where participants uncounsciously mimic a confederate's movements [42], is increased when an interdependent self-construal is primed whereas it is decreased when a dependent self-construal is activated [41]. As such, it would appear that “self-construals have a profound impact on the way people perceive others, their environment, and others” [41]. Relevant to the present discussion, it has been found that women have a predominantly interdependent self-construal whereas men have an independent self-construal [39], [43], suggesting that behaviors presumably underlied by the MNS are influenced by gender and self-concept. The fact that gender differences in self-construals have been found to be contextually dependent and variable [43] may explain why gender differences have not been reliably found in past studies. The relationship between MNS function and self-construals remains highly speculative, however, given the limited and resticted nature of the effect found in the current study. Further research is needed to specifically address whether self-concept is related to neurophysiological responses associated with the MNS.

Although the present report focuses on primary motor cortex, other brain regions have been associated with self/other representation and the judgement of similarity. For example, the medial prefrontal cortex (mPFC) has been shown to respond differentially when mentalizing about another person that is perceived to be similar or dissimilar to self [44]. The ventral mPFC appears to be associated with mentalizing about *similar* others whereas the dorsal part of the mPFC is preferentially activated when participants judge the mental state of *dissimilar* others [44]. It is interesting to note that the level of mPFC activity correlates with ratings of self/other similarity only in mentalizing tasks, such that physical judgements about another person do not specifically activate the mPFC [45]. This is in line with the suggestion that midline cortical structures are involved in “self-referential processing and understanding of others' mental states” whereas structures associated with the MNS support the physical aspects of self/other representation [32]. Another neural substrate of self representation at the “body” level is the inferior parietal cortex. The involvement of parietal areas in body schema has been reported in numerous lesion and imaging studies (see [46]). Additionnally, disruption of the inferior parietal lobule with repeated TMS impairs self-face recognition [14]. Taken together, these data suggest the existence a complex network of brain areas subtending self/other representation and the judgement of similarity at the conceptual and physical levels.

In summary, the current data show that in certain conditions motor cortex excitability is increased during the passive observation of hand movements that are physically dissimilar from the observer. Specifically, an effect of skin color was found in the right hemisphere of female participants. These results suggest that self-concept and the mirror neuron system interact at the motor cortex level in a complex and limited fashion.
